# Analysis of diffuse scattering in electron diffraction data for the crystal structure determination of Pigment Orange 13, C_32_H_24_Cl_2_N_8_O_2_

**DOI:** 10.1107/S2052520623000720

**Published:** 2023-02-24

**Authors:** Tatiana E. Gorelik, Sàndor L. Bekő, Jaroslav Teteruk, Winfried Heyse, Martin U. Schmidt

**Affiliations:** a Ulm University, Central Facility of Electron Microscopy, Materials Science Electron Microscopy, Albert Einstein Allee 11, 89069 Ulm, Germany; b Helmholtz Centre for Infection Research (HZI), SFPR, Inhoffenstraße 7, 38124 Braunschweig, Germany; c Helmholtz Institute for Pharmaceutical Research Saarland (HIPS), MINS, Campus E8.1, 66123 Saarbrücken, Germany; d Goethe University, Institute of Inorganic and Analytical Chemistry, Max-von-Laue-Str. 7, 60438 Frankfurt am Main, Germany; e Sanofi, R&D / PDP / TIDES Analytical Sciences, Building H770, 65926 Frankfurt am Main, Germany; University of Antwerp, Belgium

**Keywords:** 3D electron diffraction, electron crystallography, diffuse scattering, stacking disorder, lattice-energy minimization

## Abstract

The crystallographic study of two polymorphs of the industrial pyrazolone Pigment Orange 13 (P.O.13) is reported. The crystal structure of the β phase was determined using single-crystal X-ray analysis of a tiny needle. The α phase was investigated using three-dimensional electron diffraction. The electron diffraction data contain sharp Bragg reflections and strong diffuse streaks, associated with severe stacking disorder. The structure was solved by careful analysis of the diffuse scattering, and similarities of the unit-cell parameters with the β phase. The structure solution is described in detail and this provides a didactic example of solving molecular crystal structures in the presence of diffuse scattering. Several structural models were constructed and optimized by lattice-energy minimization with dispersion-corrected DFT. A four-layer model was found, which matches the electron diffraction data, including the diffuse scattering, and agrees with X-ray powder data. Additionally, five further phases of P.O.13 are described.

## Abstract 2

The crystal structure of the α phase of Pigment Orange 13, used until the end of 2013 to print the front covers of *Acta Crystallographica Section C* and *inter alia*, was solved by analysis of diffuse scattering in electron diffraction data.

## 1. Introduction

### 1.1. 3D electron diffraction and stacking disorder

In recent years, electron diffraction (ED) has fast gained popularity, with the development of techniques for three-dimensional (3D) data acquisition and processing (Gruene & Mugnaioli, 2021 ▸). 3D ED has been applied to almost all classes of materials, including nanocrystalline organic compounds (Gemmi *et al.*, 2019 ▸). Many striking results were obtained for crystal structures, which could not be tackled by other diffraction techniques, such as single crystal X-ray analysis or X-ray powder diffraction. In most cases, the failure of the X-ray methods was associated with (i) small crystal size, (ii) minor amount of material, (iii) polyphasic samples or (iv) severe disorder. The most difficult situations are faced when several of these issues are present simultaneously, *e.g.* a disordered nanocrystalline non phase-pure sample.

Disorder has a significant influence on the physical properties of materials, and is therefore of fundamental importance for understanding these properties (Tong *et al.*, 2015 ▸). Despite being often associated with inorganic materials, disorder is a frequent phenomenon in molecular crystals. In most cases, disorder affects side groups, such as *tert*-butyl or CF_3_ (see *e.g.* Yennawar *et al.*, 2018 ▸), or solvent molecules (see *e.g.* Spek, 2015 ▸). Stacking faults are quite frequent in organic crystals, too. Examples include compact molecules such as tris­(bi­cyclo­[2.1.1]hexeno)benzene (Bürgi *et al.*, 2005 ▸; Schmidt & Glinnemann, 2012 ▸), planar aromatic compounds such as the industrial hydrazone pigment Pigment Red 170 (Warshamanage *et al.*, 2014 ▸; Teteruk *et al.*, 2014 ▸), the pentacyclic pigment α^II^-quinacridone (Gorelik *et al.*, 2016 ▸), and the pyramid-shaped chloro(phthalo­cyaninato)­aluminium (Czech *et al.*, 2017 ▸). An unusual example is given by eniluracil, which produces a wide range of disordered structures with significant variability in physical properties, which mimics polymorphism (Copley *et al.*, 2008 ▸). Disorder of supra-molecular columns for compact disk-like molecules have been reported (Schmidt & Neder, 2017 ▸; Zehe *et al.*, 2017 ▸).

The appeal of stacking disorder lies in the ease of its detection, because any faulted sequence of layers produces streaks of diffuse scattering in reciprocal space (Welberry, 2004 ▸). If such diffuse streaks are observed in a diffraction pattern, there are three possible approaches to treat the data:

(*a*) Complete neglect of the diffuse scattering in structure solution and refinement leads to the average structure. Generally, the average structure contains a superposition of different possible atomic positions in a unit cell. For a compound with stacking disorder, the unit cell of the average structure is frequently too small to be chemically sensible, as it contains the overlay of two (or more) possible configurations.

(*b*) Evaluation of the diffuse scattering intensities at the positions of the Bragg reflections leads to an (ordered or disordered) crystal structure, which is generally chemically sensible and provides a good model for the actual structure. Supercells are used to assign side peaks along diffuse streaks as Bragg positions.

(*c*) Evaluation of the full diffuse scattering provides information on the real structure, including stacking probabilities, preferred local arrangements, deviation from the average structure *etc*. This approach is tedious, yet has been already applied to several organic compounds (see *e.g.* Weber & Bürgi, 2002 ▸; Welberry, 2004 ▸; Bürgi *et al.*, 2005 ▸; Weber & Simonov, 2012 ▸; Schmidt & Glinnemann, 2012 ▸; Teteruk *et al.*, 2014 ▸; Welberry & Weber, 2016 ▸).

For the evaluation of the diffuse scattering of tiny sub-micron organic crystals, 3D ED is the method of choice. The electron beam can be focused down to very small areas, so even very tiny crystals can be addressed individually. The scattering power of electrons is much higher than that of X-rays, hence, reasonable quality diffraction data can be obtained, despite the small crystal size. There are different flavours of experimental setup for a 3D ED experiment, including selected area ED (SAED), nanodiffraction, static patterns, precession electron diffraction, combined beam tilt/stage tilt, and continuous rotation. An overview of the different methods is given by Gemmi & Lanza (2019 ▸) and Gruene & Mugnaioli (2021 ▸). Diffraction patterns obtained with precession or continuous rotation represent a physical integration of a certain wedge of the reciprocal space within each frame. The wedge-integrated data are believed to provide a better data quality for structure analysis, compared to static, sequentially collected patterns. A special case is the analysis of electron diffuse scattering (Krysiak *et al.*, 2018 ▸, 2020 ▸; Mugnaioli & Gorelik, 2019 ▸): here, a wedge integration with a relatively large step, either with precession or continuous rotation would smear the data, and the fine details of the intensity distribution within the diffuse streaks would be lost. Therefore, for the analysis of diffuse scattering, static sequentially collected patterns are usually used (Kolb *et al.*, 2019 ▸).

In this paper we use 3D ED to determine the crystal structure of the α phase of Pigment Orange 13, which shows stacking disorder with strong diffuse scattering.

### 1.2. Pigment Orange 13

**Figure 1:**
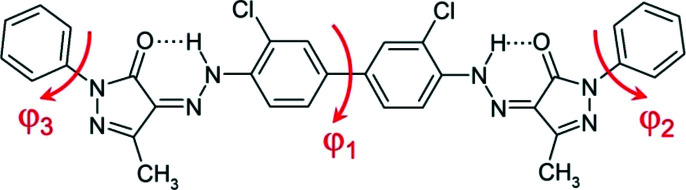
Chemical structure of Pigment Orange 13. The red arrows denote the torsion angles discussed in the text.

The molecular formula of Pigment Orange 13 (P.O.13) is shown in Fig. 1 ▸. P.O.13 is an organic hydrazone pigment. Formerly, hydrazone pigments were called ‘azo pigments’, because they were believed to contain an azo moiety (—N=N—C). However, all spectroscopic investigations and single-crystal structure analyses show that these compounds actually adopt the hydrazone-tautomeric form with a —NH–N=C group in the solid state (see *e.g.* Mustroph, 1987 ▸; Whitaker, 1988*a* ▸,*b* ▸; Ivashevskaya *et al.*, 2009 ▸; Kamei *et al.*, 2011 ▸). Hence, they must be named hydrazone pigments instead of azo pigments (Paulus, 1982 ▸; Hunger & Schmidt, 2018 ▸).

**Figure 2:**
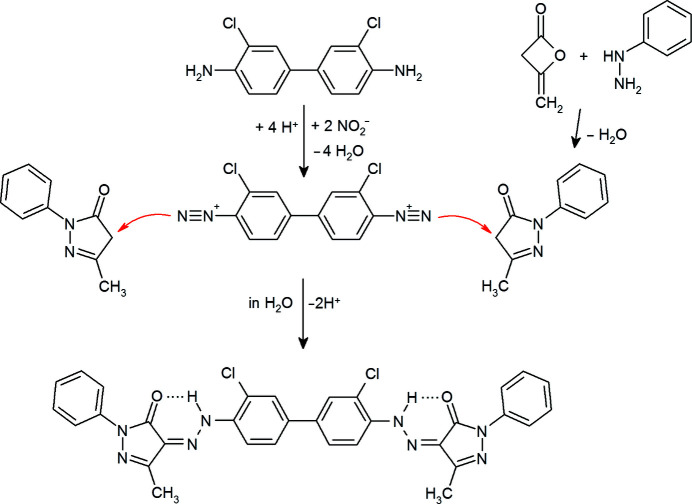
Industrial synthesis of P.O.13.

P.O.13 was invented in 1910 by A. L. Laska in the Chemische Fabrik Griesheim-Elektron in Frankfurt am Main (Laska, 1910 ▸, 1911 ▸). It has been industrially produced for more than 80 years. Its old name was ‘Vulcan Orange G extra’ (Ershov *et al.*, 1934 ▸). Later it was sold, for example, as ‘Permanent Orange G’ by Hoechst and Clariant. P.O.13 is industrially synthesized from 3,3′-di­chloro­benzidine and 5-methyl-2-phenyl-3-pyrazolone, see Fig. 2 ▸.

Most organic pigments show polymorphism (Hunger & Schmidt, 2018 ▸). However, no polymorphs were known for P.O.13 until recently. A few years ago, we performed an extensive polymorph screening, and found seven crystal phases (Bekö *et al.*, 2014 ▸). The synthesis results in the α phase. The thermodynamically more stable β phase is obtained from the α phase by recrystallization in chloro­benzene, 1,2-di­chloro­benzene or 1,2,4-tri­chloro­benzene. Five other phases (δ, ɛ_1_, ɛ_2_, ζ, η) are formed by recrystallization from other solvents, *e.g.* morpholene, dioxane, 1-chloro­naphthalene, or H_2_SO_4_ (Bekö *et al.*, 2014 ▸).

The α phase is commercially used for printing applications such as printings of packaging. For example, we found P.O.13 in Barilla noodle packaging, and in front covers of *Acta Crystallographica Section C* and *Zeitschrift für Kristallographie*, with laser-desorption-ionization mass spectrometry. P.O.13 is also used as a shading component in printing inks to give yellow pigments a warmer, light orange shade.

Like all pigments, P.O.13 is insoluble in its application medium (*e.g.* printing ink), being finely dispersed. Particle sizes are typically 50–200 nm. The crystal structures are maintained, and the resulting optical properties strongly depend on the polymorphic form and on the particle size. For example, the β phase of P.O.13 is more opaque and has a slightly more reddish shade than the α phase.

Despite the commercial importance of P.O.13, and despite its long history – P.O.13 is one of the oldest organic pigments – the crystal structures of the compound have never been revealed, hitherto.

In the following, we describe the determination of the crystal structure of the β phase of P.O.13 by single-crystal X-ray diffraction. With the knowledge of this structure, we were able to solve the crystal structure of the commercial α phase of P.O.13 by 3D electron diffraction, including the analysis of the diffuse scattering, supported by lattice-energy minimization. Additionally, we determined the crystal structure of the ɛ_1_ phase by single-crystal X-ray diffraction.

## 2. Experimental

### 2.1. Synthesis and recrystallization

P.O.13 was synthesized by diazo­tation of 3,3′-di­chloro­benzidine and subsequent coupling with 5-methyl-2-phenyl-3-pyrazolone in water, according to Fig. 2 ▸, as described by Bekö *et al.* (2014 ▸), resulting in an orange powder of the α phase.

A sample of the α phase with improved crystallinity was obtained by treatment with 2,5-hexane­dione. This sample was used for electron diffraction experiments.

The β phase was obtained by the following procedure: P.O.13 (50 mg) was dissolved in boiling chloro­benzene (or 1,2-di­chloro­benzene, or 1,2,4-tri­chloro­benzene) (20 ml), and re-precipitated by slow cooling to room temperature. The precipitate was isolated by filtration and dried at room temperature.

Single crystals of the β phase could be grown following different routes: by recrystallization from *e.g.* 1,2-di­chloro­benzene, 1,2,4-tri­chloro­benzene or amino­ethanol/butanone:

(*a*) P.O.13 (80 mg) was suspended in amino­ethanol (3 ml) using an ultrasound bath. The undissolved portion was removed by filtration. The solution was transferred into a small vial, which was closed using a lid with a pinhole, and put into a flask containing butanone (7 ml) as an anti-solvent. The flask was sealed and kept at room temperature. The butanone slowly diffused via the gas phase into the amino­ethano­lic solution causing P.O.13 to precipitate as needle-shaped single crystals of the β phase.

(*b*) P.O.13 (50 mg) was dissolved in boiling 1,2,4-tri­chloro­benzene (20 ml; b.p. 213°C). After one hour, the solution was allowed to cool slowly to room temperature, resulting in small block-like single crystals of the β phase.

(*c*) The same as (*b*), but with 150 mg of P.O.13, giving crystals of the β phase of P.O.13 in the shape of elongated plates.

The γ phase does not exist. The samples which we initially denominated as γ phase turned later out to be mixtures of other phases.

The δ phase was obtained by recrystallization from amino­ethanol/dioxane: α-P.O.13 (50 mg) was dissolved in 2-amino­ethanol (3 ml). 1,4-Dioxane (7 ml) was slowly added, and the mixture was stored for 5 days at room temperature. The precipitate was isolated by filtration and dried at ambient conditions.

Single crystals of the ɛ_1_ phase were obtained by crystallization from amino­ethanol/dioxane: α-P.O.13 (80 mg) was suspended in amino­ethanol (3 ml) using an ultrasound bath for 5 min at room temperature. The remaining solid was removed by filtration. The filtrate was transferred into an open vial, which was placed together with dioxane (7 ml) in a closed flask, and kept at room temperature. The dioxane slowly diffused via the gas phase into the amino­ethanol, causing the formation of orange needle-shaped single crystals of the ɛ_1_ phase with dimensions of about 0.1 mm × 0.01 mm × 0.01 mm.

The ɛ_2_ phase was obtained by crystallization from morpholene: P.O.13 (60 mg) was heated with morpholene (20 ml; b.p. 129°C) to reflux. The solid was isolated by hot filtration, and dried at room temperature. The resulting powder contained tiny thin needles (probably ɛ_2_ phase), which were too small for single-crystal X-ray diffraction. The powder pattern showed a mixture of phases β, δ and ɛ_2_.

The ζ phase was formed by recrystallization of α-P.O.13 (100 mg) in 1-chloro­naphthalene (5 ml) at 130°C and slow cooling to room temperature. The pigment was filtrated, and dried at room temperature. A pure ζ phase was obtained only once; reproduction attempts led to mixtures of ζ and β phases, or to the pure β phase.

The amorphous η phase was obtained from concentrated H_2_SO_4_: α-P.O.13 (40 mg) was dissolved in concentrated sulfuric acid (3 ml) at room temperature in an open vial. The open vial was placed in a larger vial, and surrounded by water (7 ml). The larger vial was sealed. The water slowly diffused into the H_2_SO_4_ solution, causing the pigment to precipitate. The precipitate was filtered, washed with water and dried at room temperature.

### 2.2. Sample characterization

#### 2.2.1. Methods

All crystalline phases were characterized by powder X-ray diffraction, thermal, spectroscopic and elemental analyses.

X-ray powder patterns were recorded in transmission mode on a Stoe Stadi-P diffractometer equipped with a Ge(111) monochromator and a position-sensitive detector using Cu *K*α_1_ radiation. The sample was rotated during the measurement.

Thermogravimetric analysis (TGA) was carried out using a TGA 92 system (SETERAM Instrumentation). Differential scanning calorimetric (DSC) measurements were performed with a DSC 131 systems (SETERAM Instrumentation) in the temperature window from room temperature up to 500°C.

Spectroscopically the samples were characterized by FTIR using a Shimadzu FTIR-8300 measuring in transmission mode; LDI-TOF-MS using a Voyager-DE STR (Applied Biosystems Inc.) using a nitro­gen laser with 337 nm and 10 ns; all liquid ^1^H NMR studies were carried out using an Avance 250 MHz NMR spectrometer (Bruker) at 300 K in *d_2_*-sulfuric acid.

Elemental analyses (EA, only C:H:N quantification) were performed on a vario MICRO cube from Elementar Analytical Systems GmbH.

#### 2.2.2. Analytical data of individual phases

The α phase resulting from synthesis and crystallization experiments shows one single weight loss in the TGA starting at 305°C, and a signal in the DSC resulting from decomposition at approx. 324°C, which points to a solvent-free phase. FTIR and ^1^H NMR showed the typical signals and the LDI-TOF-MS the typical fragmentation signals at the NH–N bond on one or both sides of the molecule. Furthermore, the FTIR confirmed the bis­(hydrazone)-tautomeric form. The solvent-free character could be confirmed by EA calculated for P.O.13 C_32_H_24_Cl_2_N_8_O_2_ (%): C 61.64, H 3.88, N 17.97; found: C 61.42, H 3.65, N 18.30.

The β phase exhibits a similar behaviour in thermal analyses as the α phase. The mass loss starts at 305°C in the TGA. The decomposition starts at 311°C, pointing again to a solvent-free phase. In the FTIR and ^1^H NMR spectra, no significant differences to the α phase could be observed. The EA confirmed the solvent-free form with calculated values for P.O.13 C_32_H_24_Cl_2_N_8_O_2_ (%): C 61.64, H 3.88, N 17.97; found: C 59.43, H 3.25, N 16.67.

For the δ phase very similar thermal analytical results could be found as for the α and β phases. Here again a solvent-free phase could be found, which was also confirmed by EA with calculated values of P.O.13 C_32_H_24_Cl_2_N_8_O_2_ (%): C 61.64, H 3.88, N 17.97; found: C 60.41, H 3.91, N 17.73.

The ɛ_1_ and ɛ_2_ phases occurred only in a mixture with the δ phase. The ɛ_1_ phase could only be analysed using X-ray powder diffraction and single-crystal X-ray diffraction, the ɛ_2_ phase only by X-ray powder diffraction.

The ζ phase showed an IR spectrum is similar to that of the other phases. The ^1^H NMR showed additional peaks, which could not be attributed to P.O.13 or 1-chloro­naphthalene. The reason is unknown (by-products from the synthesis?). TGA revealed a mass loss of 4.8% between 120°C and 180°C, which was absent in the other solvent-free phases, and might come from 1-chloro­naphthalene (b.p. 260°C) absorbed at the surface of the powder. Melting under decomposition was observed at 321°C.

The η phase showed similar thermal and spectroscopical results as the other solvent-free forms. EA data: calculated for P.O.13 C_32_H_24_Cl_2_N_8_O_2_ (%): C 61.64, H 3.88, N 17.97; found: C 59.21, H 3.89, N 17.01. The deviation of calculated and found values may be caused by remaining amounts of water or H_2_SO_4_.

### 2.3. Electron diffraction of the α phase

Samples for TEM investigations were prepared by ultrasonication in *n*-hexane. A drop of the obtained suspension was placed onto a carbon-coated copper grid (Plano, Wetzlar, S160) and dried in air.

The TEM studies were carried out using a TECNAI F30 TEM (Thermofisher, The Netherlands) equipped with a field emission gun running at 300 kV. Electron diffraction data were collected using an automated acquisition module working in STEM / nano-diffraction modes, as described earlier (Kolb *et al.*, 2007 ▸). The beam diameter for the nano-diffraction measurements was 100 nm. Diffraction frames were collected sequentially through the goniometer tilt step of 1°. No electron beam precession was used. Four electron diffraction tilt series were collected with the total tilt range between 50° and 86° (Table 1 ▸).

The data were processed using *EDT Process* software (AnaliteX, Sweden), supported by self-written MatLab scripts. Visualization of the reconstructed diffraction volumes were produced using the UCSF *Chimera* package (Pettersen *et al.*, 2004 ▸). Sections of the reciprocal space representing the main crystallographic zones were calculated in *PETS2* (Palatinus *et al.*, 2019 ▸).

Electron diffraction patterns of the obtained models were kinematically simulated using *eMAP* (AnaliteX, Sweden) software.

### 2.4. Single-crystal X-ray structure analyses of the β and ɛ_1_ phases

#### 2.4.1. β phase

The crystal structure of a tiny needle was determined using a Bruker SMART three-circle diffractometer equipped with a copper Incoatec IμS microfocus X-ray source and an APEX2 CCD detector. The structure was solved by direct methods with *SHELXS97* (Sheldrick, 1990 ▸) and refined with *SHELXL97* (Sheldrick & Schneider, 1997 ▸). All non-H atoms were refined anisotropically.

The structure of the β phase was originally solved with the unit cell setting *a* = 14.425 (4) Å, *b* = 12.127 (3) Å, *c* = 33.137 (9) Å, space group *Pbca*, *Z* = 8. To facilitate the comparison of the α and β phases, we transformed the unit cell with ***a*** ′ = ***c***, ***b*** ′ = ***a***, ***c*** ′ = ***b***, resulting in the unit-cell parameters *a* = 33.137 (9) Å, *b* = 14.425 (4) Å, *c* = 12.127 (3) Å. The space group remained to be *Pbca*, *Z* = 8. The latter unit cell setting is used throughout this paper (Table 2 ▸).

#### 2.4.2. ɛ_1_ phase

The crystal structure of the ɛ_1_ phase (dioxane disolvate monohydrate) was determined by single-crystal X-ray diffraction as described for the β phase. The data quality was very limited. The dioxane molecule is severely disordered. The scattering power of the dioxane molecule (C_4_H_8_O_2_, in total 48 electrons) was approximated by eight carbon atoms (48 electrons). The water molecule was approximated by a single oxygen atom.

### 2.5. Generation of ordered structural models

Ordered and disordered structural models were constructed using the software *Materials Studio* (Version 4.4, BIOVIA Dassault Systèmes, San Diego, USA).

### 2.6. Lattice-energy minimization

Preliminary lattice-energy minimizations were carried out by force-field methods using the *Materials Studio* software. The Dreiding force-field (Mayo *et al.*, 1990 ▸) was combined with atomic charges calculated by the Gasteiger method (Gasteiger & Marsili, 1980 ▸).

The lattice-energy minimizations described in this paper were performed with dispersion-corrected density functional theory (DFT-d) using the program *GRACE* (Neumann *et al.*, 2008 ▸). *GRACE* uses the VASP code (Kresse & Hafner, 1993 ▸; Kresse & Furthmüller, 1996*a* ▸,*b* ▸; Kresse & Joubert, 1999 ▸) for the DFT calculations. The PBE functional was applied. For the van der Waals interactions, an empirical dispersion correction was used (Neumann & Perrin, 2005 ▸).

Two sets of calculations were performed: (*a*) optimization of the atomic coordinates, with unit-cell parameters fixed to the values determined from electron diffraction, (*b*) optimization of the unit-cell parameters together with the atomic coordinates.

## 3. Results and discussion

### 3.1. Crystal structure of the β phase, serving as the basis for the structure solution of the α phase

**Figure 3:**
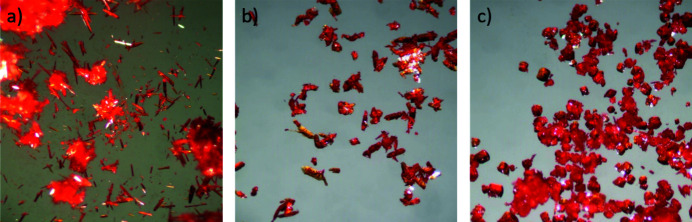
Different morphologies of P.O.13, β phase: (*a*) needles from amino­ethanol/butanone; (*b*) elongated plates from recrystallization in 1,2,4-tri­chloro­benzene with higher concentration of P.O.13; (*c*) blocks from recrystallization in 1,2,4-tri­chloro­benzene.

Recrystallization of P.O.13 resulted in single crystals with different morphologies: needles, platelets and blocks, see Fig. 3 ▸.

All crystals were rather small, so initially all attempts to determine the structure from single-crystal X-ray diffraction failed. Different diffractometers were tried. Finally, the crystal structure of a needle with a size of 0.050 mm × 0.050 mm × 0.3 mm could be determined at Sanofi (Frankfurt-Höchst, Germany), using a diffractometer equipped with a microfocus X-ray source and a CCD detector.

Despite the different morphologies, all crystals shown in Fig. 3 ▸ correspond to the same β phase, as proven by single-crystal X-ray analyses and X-ray powder diffraction.

The β phase crystallizes in the space group *Pbca* with unit-cell parameters of *a* = 33.137 (9), *b* = 14.425 (4), *c* = 12.127 (3) Å, α = β = γ = 90°, *V* = 5797 (3) Å^3^. Crystallographic data are given in Table 2 ▸.

The crystal structure analysis proves that P.O.13 possesses the hydrazone tautomeric form, not the azo form, in the solid state – like it was observed for all other industrial organic pigments, too.

**Figure 4:**
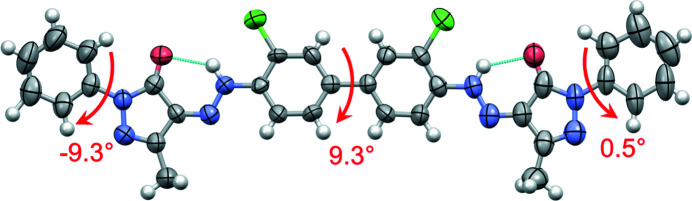
Molecular structure of P.O.13 in its β phase. Ellipsoids drawn with 50% probability, H atoms with arbitrary radius. The central bi­phenyl system adopts a *cis* conformation. The arrows denote the interplanar angles between the central phenyl rings, and between the terminal phenyl and pyrazole rings.

The molecular conformation of P.O.13 in its β phase is shown in Fig. 4 ▸. The two phenyl rings in the centre of the molecule are almost coplanar and form a dihedral angle of φ_1_ = 9.3°. In the gas phase or in solution, the central bi­phenyl fragment is twisted by about 40°. Hence, the planarity of the bi­phenyl fragment is a packing effect, because planar molecules can generally adopt a more efficient packing with a higher packing energy (Schmidt *et al.*, 2007 ▸). Several other bi­phenyl-hydrazone pigments show a planar conformation, too.

**Figure 5:**
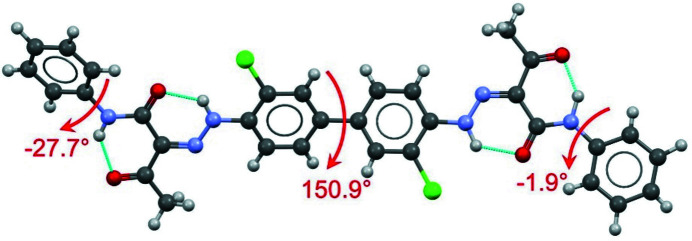
Molecular *trans* conformation found in all other hydrazone pigments based on 3,3′-di­chloro­benzidine. Here: α phase of Pigment Yellow 12. The values denote the dihedral angles (Barrow *et al.*, 2000 ▸).

An isolated molecule of P.O.13 can rotate around the central phenyl–phenyl bond, so it can adopt an overall *cis* conformation (*i.e.* with chlorine atoms on the same side of the molecule) or an overall *trans* conformation (with Cl on opposite sides). Both conformations have a similar intramolecular energy. Astonishingly, the β phase of P.O.13 shows the molecule in an overall *cis* conformation, see Fig. 4 ▸. In contrast, all other known crystal structures of hyrazone pigments based on 3,3′-di­chloro­benzidine exhibit the *trans* conformation, with torsion angles between 151.9 and 180°, see Fig. 5 ▸ (Barrow, 2002 ▸; Barrow *et al.*, 2000 ▸, 2003 ▸; Schmidt *et al.*, 2007 ▸). The reason why P.O.13 adopts a *cis* conformation in its β phase is unknown. Apparently, it is a packing effect, but details remains obscure.

The terminal phenyl rings are slightly rotated relative to the adjacent pyrazolone moieties, with torsion angles of 0.5 and −9.3°, respectively. In other pyrazolone pigments, this torsion angle varies between 0° and 35° (see Figs. S1 and S2).

**Figure 6:**
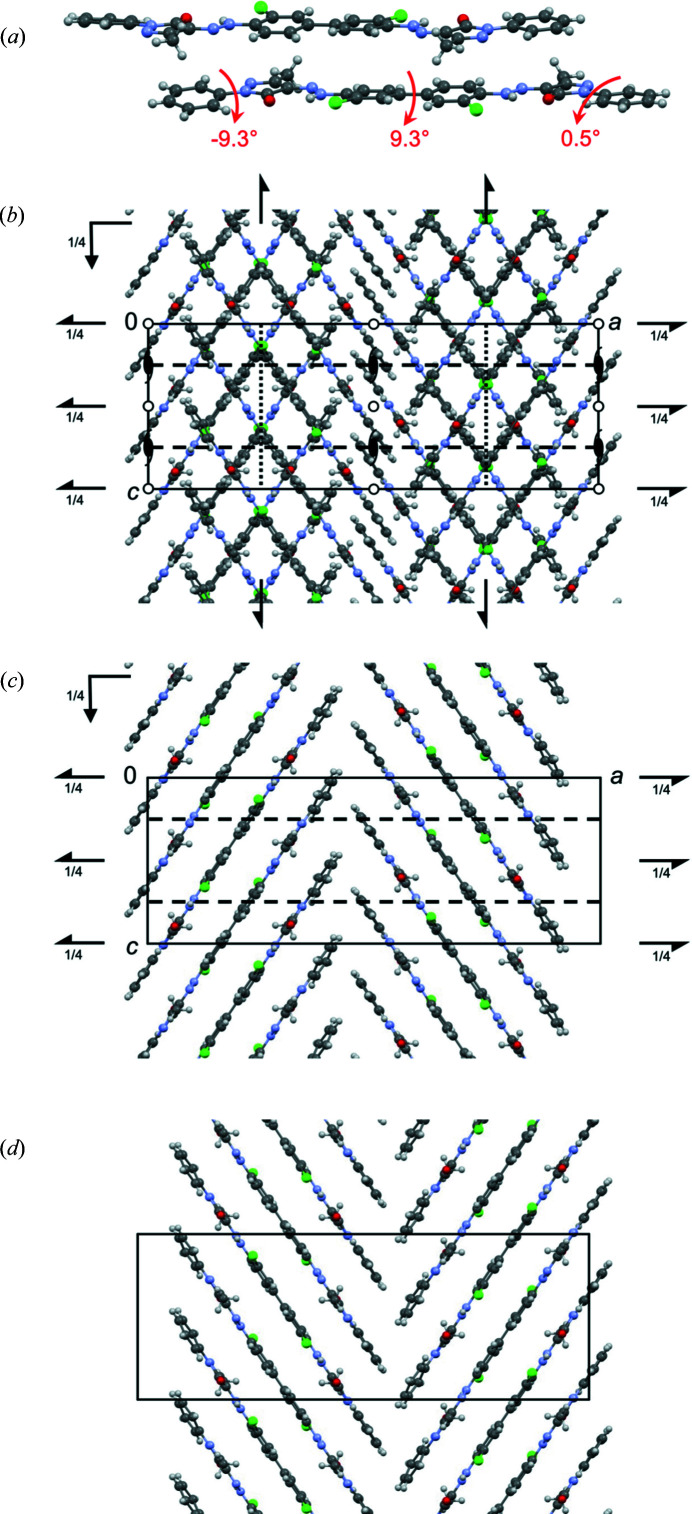
Crystal structure of the β phase of P.O.13 (in space group *Pbca*). (*a*) Mutual arrangement of two neighbouring molecules. The arrows denote the interplanar angles. View direction ≈ [801]. The two molecules are related through a *c* glide plane. (*b*) Packing diagram, with symmetry elements. View direction [010]. (*c*) First molecular layer, containing the molecules in the range 0 < *y* < 0.5, with layer symmetry elements. All symmetry elements are crystallographic ones. View direction [010]. (*d*) Second layer, containing molecules in the range 0.5 < *y* < 1. View direction [010].

In β-P.O.13, the molecules are stacked on top of each other (Fig. 6 ▸). The stacking results in a dense packing with a favorable lattice energy, which would not be possible for twisted molecules.

In the β phase, the unit cell contains two layers running parallel to (010). In each layer, the molecules form a herringbone arrangement, see Figs. 6 ▸(*c*) and 6 ▸(*d*). The layers are stacked along the *b* axis.

### 3.2. Electron crystallography of the α phase

#### 3.2.1. Crystal morphology

No crystals of the α phase with a size suitable for single-crystal X-ray diffraction could be grown. The X-ray powder diffraction diagram consists of only a few peaks (see Section 3.2.12), which could not be reliably indexed. Therefore, single-crystal 3D ED data were collected.

**Figure 7:**
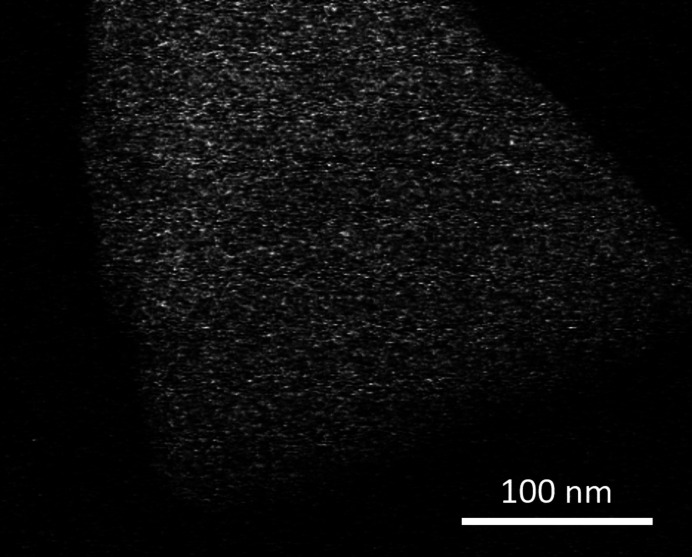
STEM image of an α-P.O.13 crystal.

Electron microscopy revealed that the crystals of α-P.O.13 have an irregular shape with sizes ranging from 200 nm to 1.5 µm. A STEM image of a crystal is shown in Fig. 7 ▸. Despite the absence of well defined facets, all studied crystals had the same orientation on the TEM grid. Consequently, one dimension of the crystals (along the electron beam, normal incidence) is apparently significantly shorter than the others. This direction was taken as [100] direction of the crystal structure.

#### 3.2.2. 3D electron diffraction

**Figure 8:**
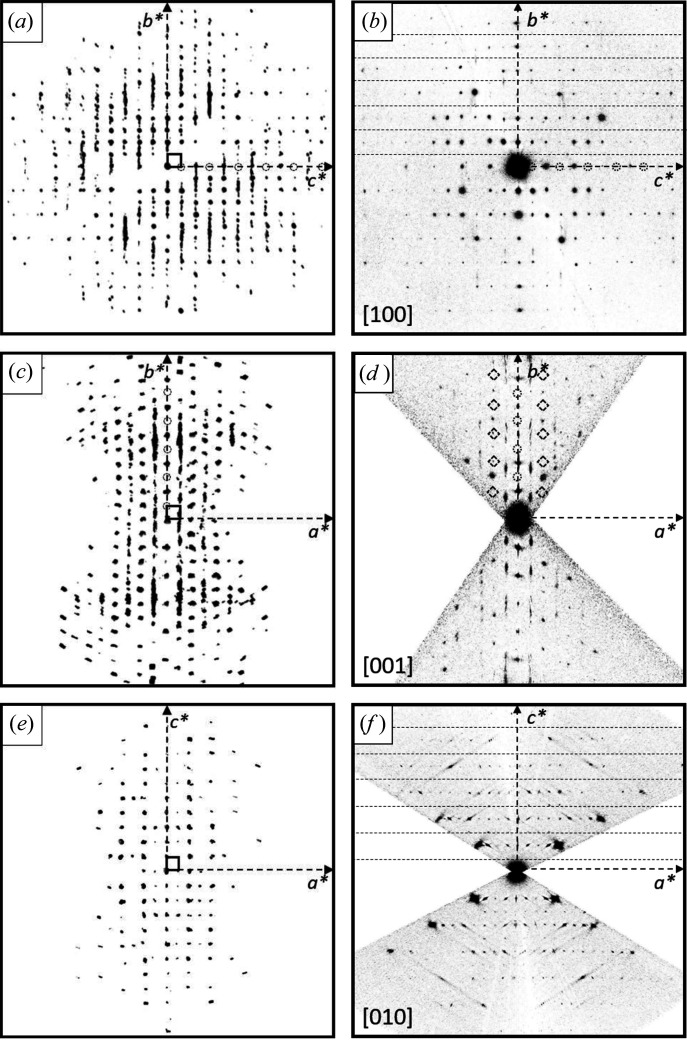
Three-dimensional electron diffraction patterns of α-P.O.13. The projections of the reciprocal volume are shown on the left hand side; the cuts through the reciprocal space representing the main crystallographic zones are shown on the right side. The small square denotes the reciprocal unit cell of the original unit-cell setting, which is discussed here. (*a*) View of the reciprocal space along a*; empty circles mark rows of missing *h*0*l* reflections for odd l. (*b*) [100] zone pattern as a section from the reciprocal space; dashed lines mark rows of extinct reflections with the reflection condition 0*kl*: *k* = 2*n*; empty circles mark rows of missing *h*0*l* reflections, as in (*a*). (*c*) View of the reciprocal space along c*. The empty circles mark rows of missing reflections in the 0*kl* zone with reflection condition: *k* = 2*n*. (*d*) [001] zone as a slice from the reciprocal volume. Empty circles denote missing 0*k*0 reflections. The diamonds mark extinct reflections, which correspond to the unit cell of the average structure with *a*_av_* = 2a* with the reflection condition *h*_av_ *k*_av_0: *h*_av_ + *k*_av_ = 2*n*. (*e*) View of the reciprocal volume along b*. (*f*) [010] zone as a slice from the reciprocal space; dashed lines represent extinctions of every second line in the *h*0*l* plane with the reflection condition *l* = 2*n*. A possible extinction of *h*00 reflections is not visible, due to the missing cone of reflections parallel of the electron beam.

3D ED datasets of four different crystals were collected. For all datasets, initially an orthorhombic unit cell with *a* ∼ 16 Å, *b* ∼ 14 Å and *c* ∼ 12 Å was found. The main projections of the reconstructed 3D reciprocal space as well as the main crystallographic zones (obtained by a corresponding cut of the reciprocal space) are shown in Fig. 8 ▸.

The diffraction patterns of all investigated crystals consist of a mixture of discrete Bragg reflections and strong parallel streaks of diffuse scattering. Such parallel rods of diffuse scattering are typical for crystals with stacking disorder, which are often observed for layered materials (Welberry, 2004 ▸).

In layered structures, frequently the interactions between the layers are much weaker than the bonding within a layer. Correspondingly, the crystals grow preferably along the layer directions. In a TEM experiment, in which the crystals are placed onto a flat support normal to the incident electron beam, the layered crystals are usually oriented with their layers parallel to the supporting film, so that the stacking direction is perpendicular to the film, parallel to the incident electron beam. In the presence of stacking faults, or stacking disorder, the associated diffuse scattering usually runs parallel to the electron beam. This situation was, for instance, observed in the electron diffraction investigations of α^II^-quinacridone (Gorelik *et al.*, 2016 ▸). In the case of α-P.O.13, the diffuse scattering is not running parallel to the electron beam, but orthogonal to it. Correspondingly, the stacking direction is not aligned along the shortest crystal dimension, but is oriented perpendicular to it. The explanation for this unusual behaviour is given in Section 3.2.15.

#### 3.2.3. Determination of unit-cell parameters

First, the unit-cell parameters were determined from electron diffraction data, taking into account the positions of the diffuse streaks. The resulting unit cell will be called the original setting of the unit cell, and denoted with the unit-cell parameters *a*_o_, *b*_o_ and *c*_o_, in order to distinguish them from other cell settings discussed later.

The unit-cell parameters of the four investigated crystals agreed well, and lead to an orthorhombic unit cell within the accepted tolerance for ED data, see Table 1 ▸. The averaged lattice parameters were *a*_o_ = 16.1 (3) Å, *b*_o_ = 14.5 (2) Å, *c*_o_ = 12.3 (1) Å.

#### 3.2.4. Systematic extinctions and the space group

The unit cell is orthorhombic primitive (Table 1 ▸). The projection of the reciprocal volume along a*, b* and c*, and the zones [100], [010] and [001] are shown in Fig. 8 ▸. In the diffraction patterns, the following reflection conditions were observed:

*hkl*: none

0*kl*: *k* = 2*n*

*h*0*l*: *l* = 2*n*

*hk*0: none

*h*00: not observable, due to the missing cone of reflections along the incident electron beam

0*k*0: *k* = 2*n*

00*l*: *l* = 2*n*.

These extinctions lead to the extinction symbol *Pbc* –, which corresponds to the space groups *Pbc*2_1_ (non-standard setting of *Pca*2_1_, No. 29), or *P* 2/*b* 2_1_/*c* 2_1_/*m* (*Pbcm*, No. 57) (Hahn, 2002 ▸). After structure solution (see below), space group *Pbcm* could be ruled out, because the molecular arrangement disagrees with the mirror plane.

#### 3.2.5. Diffuse scattering

The diffuse scattering of α-P.O.13 exhibits three main features:

(i) The diffuse scattering consists of strong diffuse streaks parallel to b* (Fig. 8 ▸). Correspondingly, the structure consists of layers parallel to (010), which exhibit a stacking disorder along **b**. Note that the expression ‘layer’ in the description of a stacking disorder does not need to be a ‘layer’ in the usual meaning for a chemist, but can be any kind of a two-dimensional building block.

(ii) The diffuse streaks are located between the sharp *hkl* Bragg reflections: layers of reflections with even *h* consist of sharp Bragg reflections without any diffuse scattering; rows with odd *h* consist of diffuse scattering only [Figs. 8 ▸(*a*), 8 ▸(*c*), 8 ▸(*d*)]. Correspondingly, the average structure, related to the Bragg reflections only, can be described by a unit cell with *a*_av_* = 2*a*_o_*, hence *a*_av_ = *a*_o_/2 ≃ 8.1 (1) Å, *b*_av_ = *b*_o_, *c*_av_ = *c*_o_, α_av_ = β_av_ = γ_av_ = 90°. This average structure has translational symmetry with a periodicity of *a*_o_/2 ≃ 8.1 (1) Å. If the actual structure is described with the original unit cell (*a*_o_, *b*_o_, *c*_o_), then each layer can adopt two positions, which differ by Δ*x* = *a*_o_/2 ≃ 8.1 (1) Å. The layers themselves are apparently ordered, since there is no indication for any additional diffuse scattering outside the streaks (except thermal diffuse scattering, which is always present).

**Figure 9:**
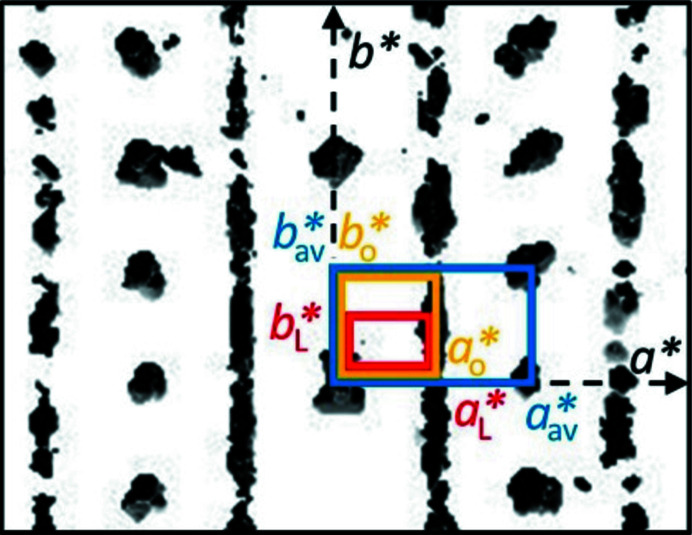
Reciprocal unit cells of α-P.O.13, used to describe the electron diffraction data. View of the reciprocal space volume along a*. The original unit cell is drawn in yellow, the large unit cell in red and the unit cell of the average structure in blue.

For the unit cell of the average structure, all reflection conditions of the original unit cell remain valid: (*hkl*: none; 0*kl*: *k* = 2*n*; *h*0*l*: *l* = 2*n*), see Fig. 8 ▸. Additionally, a new rule appears. In the zone [001] half of the reflections is extinct [marked by diamonds in Fig. 8 ▸(*d*)], corresponding to the reflection condition *hk*0: *h*+*k* = 2*n*. The combination of these extinction rules leads to the extinction symbol *Pbca*, corresponding to space group *Pbcn* (No. 60), for the average structure. Hence, the average structure has a higher symmetry than the original structure itself, which is a common phenomenon in disordered materials.

(iii) Within the diffuse streaks, there are intensity maxima at half-integer *k* values, *i.e.* at *k* = ±0.5, ±1.5, ±2.5, ±3.5 *etc*., see Fig. 8 ▸(*c*). At integer *k* values, *i.e.* at the Bragg positions, the intensity approaches minimum. To describe these maxima as Bragg peaks, the reciprocal unit cell has to be halved, *b*_L_* = *b*_o_*/2, see Fig. 9 ▸. In real space this enlarges the unit cell by 2, corresponding to the lattice parameters of *b*_L_ = 2*b*_o_ ≃ 29.1 (4) Å, *a*_L_ = *a*_o_, *c*_L_ = *c*_o_, α_L_ = β_L_ = γ_L_ = 90°, see Table 3 ▸. Hence, the preferred local structure has a repetition unit of *b*_L_ ≃ 29.1 (4) Å.

The large unit cell exhibits the following reflection conditions [Figs. 8 ▸(*c*) and 9 ▸]: *hkl*: *h*+*k* = 2*n*, which reveals the preferred local structure as being *C*-centred. Obviously, this extinction rule within the diffuse lines is not strictly followed, hence, the C-centring should be treated as an approximation only.

In addition to the strong diffuse streaks parallel to b*, the diffraction pattern shown in Fig. 8 ▸(*f*) contains faint diffuse streaks parallel to [201] and 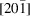 . The origin of this feature is discussed in Section 3.2.14.

#### 3.2.6. Structure determination (two-layer model)

We tried to extract the intensities of electron diffraction reflections and perform an *ab initio* structure analysis using direct methods as implemented in *SIR* (Burla *et al.*, 2015 ▸). The resulting scattering density maps could not be interpreted with a chemically sensible molecular packing of P.O.13, possibly due to the presence of the stacking disorder.

**Figure 10:**
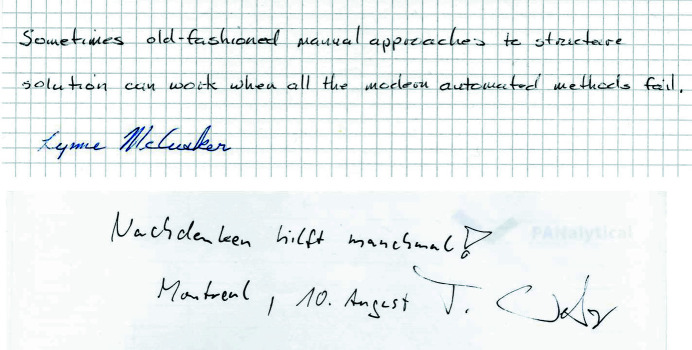
Suggestions for manual approaches of structure solution in difficult cases, collected at the IUCr conference in Montréal, 2014. Top: Lynne McCusker (ETH Zürich), bottom: Thomas Weber (ETH Zürich). Translation from German: ‘Thinking helps sometimes’.

The structure of α-P.O.13 was solved by a manual approach. Such a procedure was common in the early days of X-ray crystallography. For example, Kathleen Lonsdale solved the triclinic structure of hexa­methyl­benzene in 1929 by careful visual consideration of the distribution of individual reflection intensities in the diffraction pattern (Lonsdale, 1929 ▸). Also, in the early days of electron crystallography on organic compounds, a very similar approach was used, *e.g.*, for the structure solution of Pigment Red 53:2 (Gorelik *et al.*, 2009 ▸). Still today, the manual approach is valuable, *e.g.*, for difficult structure solutions from powder data, or for quasicrystals, see Fig. 10 ▸.

The number of molecules per unit cell was deduced from the unit cell volumes of the α and β phases, revealing that the unit cell of the α phase (with lattice parameters *a*_o_, *b*_o_, *c*_o_) contains 4 molecules of P.O.13.

The α and β phases of P.O.13 have very similar lattice parameters, with *a*_oα_ ≃ ½ *a*_β_, *b*_oα_ ≃ *b*_β_, *c*_oα_ ≃ *c*_β_, α = β = γ = 90°. Also the space groups showed some similarities (*Pbca* for the α phase, *Pbc*2_1_ or *Pbcm* for the β phase in its original unit-cell setting). These observations indicated that the crystal structure of the α phase might contain somehow similar features as the structure of the β phase.

For the structure solution of α-P.O.13, we assumed that neighbouring molecules are arranged in a similar way as in the β phase (Fig. 6 ▸). The structure was then solved manually by three of us (JT, MUS and TEG) using print-outs of the molecular packing shown in Figs. 6 ▸(*b*)–6 ▸(*d*).

**Figure 11:**
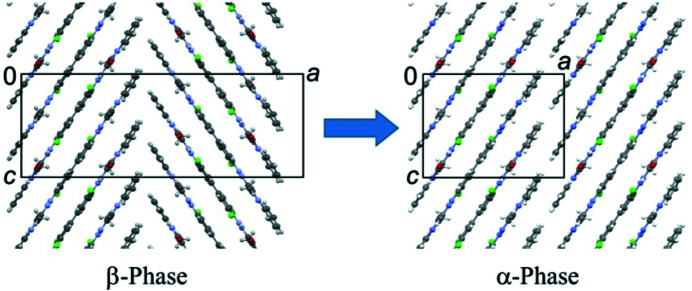
Construction of the crystal structure of the α phase from the structure of the β phase. Only the first layer is shown, with molecules in the range 0 < *y* < 0.5. View direction [010].

To account for the reduction of the unit-cell parameter *a*_0_ from 33.1 to 16.1 Å, we omitted half of the unit cell content of the β phase, see Fig. 11 ▸.

**Figure 12:**
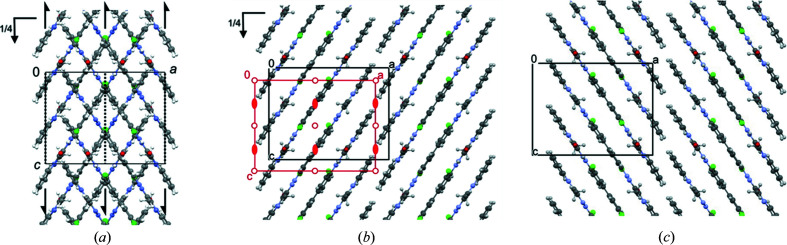
Crystal structure model of the α phase of P.O.13. Ordered two-layer model, after optimization by DFT-d. View direction [010]. (*a*) Molecular packing, with symmetry elements shown (space group *Pbc*2_1_). (*b*). First single layer (0 < *y* < 0.5, layer *A*), with symmetry elements. Unit cell and symmetry elements of the crystal structure are drawn in black. The layer group of an individual layer is *P*12/*c*1. The corresponding unit cell and symmetry elements of the single layer are drawn in red. Note that the origin of the red unit cell is located at *y* = 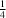 , *i.e.* in the centre of the layer. In the ordered two-layer model (*Pbc*2_1_), only the *c*-glide plane is a crystallographic (global) symmetry element, whereas 2 and 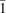 are only local ones. In the ordered four-layer model (space group *P*2_1_/*c*, see below), only the inversion centre is a global symmetry element. (*c*) Second single layer (0.5 < *y* < 1, layer *B*), being symmetrically equivalent to the first one.

The obtained structure consists of two layers in the **b** direction, like the β phase. The lattice parameter *a*_0α_ is now *a*_0α_ = ½ *a*_β_, as seen from the ED data. Upon this cell reduction, all symmetry elements of the β phase, which connected the omitted half of the unit cell with the remaining one, were lost. From the original symmetry *P* 2_1_/*b* 2_1_/*c* 2_1_/*a* (Fig. 4 ▸) only *Pbc*2_1_ remains. No additional symmetry elements were generated, apart from the translation vector of ½*a*_β_ (which also leads to a doubled density of 2_1_ and *b* symmetry elements). Consequently, the new structure has *Pbc*2_1_ symmetry, see Fig. 12 ▸(*a*). This symmetry fully matches the extinction conditions observed in the ED patterns. *Pbc*2_1_ is a non-standard setting of *Pca*2_1_ (space group No. 29).

Upon the cell reduction from the β phase to the α phase, the symmetry of a single molecular layer changes from *P*2_1_ *ca* [Fig. 6 ▸(*c*)] to *P*12/*c*1, see Fig. 12 ▸(*b*). However, the twofold axis and the inversion centre are only local symmetry elements. Only the *c*-glide plane is a global one.

This structural model is ordered (ordered two-layer model). It shows a sensible molecular geometry, reliable intermolecular contacts, and a dense molecular packing. The model was validated by lattice-energy minimization (see below). Hence, from the crystal-chemical point of view, this model is fully sensible. The unit-cell parameters from DFT-d optimization were similar to those derived from ED. The extinction conditions are fulfilled as well. However, the model does not include the stacking disorder and yet, and does not give any explanation for the diffuse scattering.

#### 3.2.7. Stacking disorder

**Figure 13:**
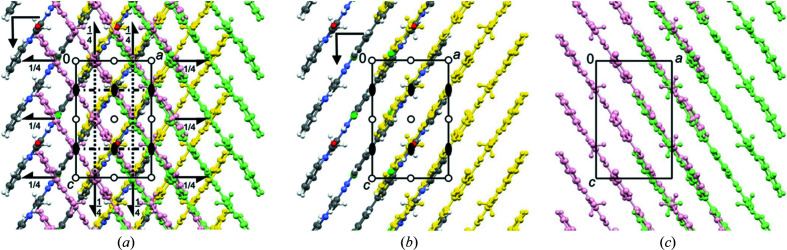
Average structure of the α phase of P.O.13. View direction [010]. (*a*) Overall structure, with symmetry elements (space group *Pbcn*). (*b*). First single layer, with symmetry elements (layer group *P*2/*c*). All symmetry elements of the layer group are crystallographic ones of *Pbcn*. Molecules in position *A* are drawn in element colours, those in position *C* in yellow. (*c*) Second single layer. Molecules in position *B* are drawn in pink, in position *D* in green.

In the ordered two-layer model, the molecules in the first layer (layer *A*) are centred at *x*_0_ = 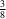 , in the second layer (layer *B*) at *x*_0_ = 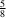 , see Figs. 12 ▸(*b*) and 12 ▸(*c*). Hence, the centre of the second layer is shifted against the centre of the first layer by *a*/4. The diffuse scattering revealed that every layer has two possible positions, which differ by ± *a*/2. Consequently, the first layer could alternatively be located at *x*_0_ = 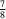 (layer *C*), and the second layer at *x*_0_ = 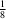 (layer *D*), see Fig. 13 ▸. In other words, each subsequent layer is shifted in respect to the preceding either by ± *a*/4. This ambiguity leads to the stacking disorder.

The superposition of both lateral positions in each layer leads to the average structure. In accordance with the ED data, this average structure can be described with a smaller unit cell with *a*_av_ = *a*_o_/2, *b*_av_ = *b*_o_ , *c*_av_ = *c*_o_. The average structure has the space group *Pbcn*, see Fig. 13 ▸. The unit cell and the systematic extinctions are, again, in perfect agreement with the electron diffraction data.

Within each layer, all molecules must have the same lateral shift, *i.e.* all molecules in the first layer must either all be at position *A*, or all at position *C*, and in the second layer either all at *B* or all at *D*. A mixture of molecular positions within a layer would result in large voids or severe molecular overlap. Hence, the layer itself is ordered, which explains the absence of diffuse scattering in other directions apart from b*.

**Figure 14:**
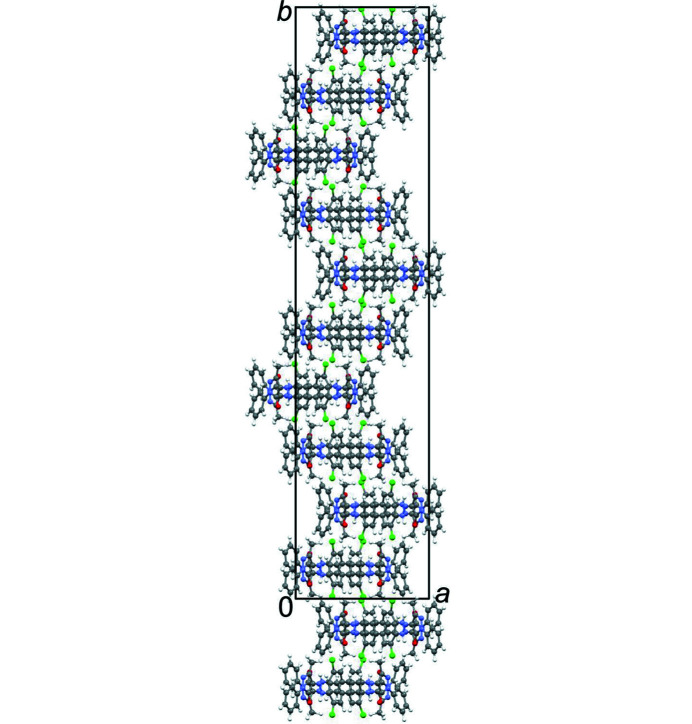
Model for a stacking sequence with ten layers, having the periodic sequence |*ABADABADAB*|. View direction [001].

There is an infinite number of possible stacking sequences, periodic and non-periodic. The simplest periodic stacking sequences are |*AB*|*AB*|*AB*| (ordered two-layer model), |*ABCB*|*ABCB*|, and |*ABCD*|*ABCD*| (the vertical lines denote the repeating unit). A stacking sequence with a periodicity unit of ten layers (|*ABADABADAB*|*ABADABADAB*|) is shown in Fig. 14 ▸.

#### 3.2.8. Preferred local structure (four-layer model)

**Figure 15:**
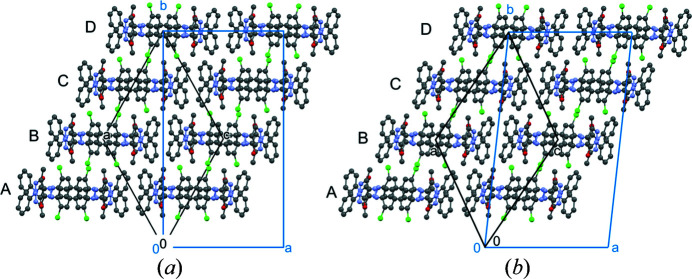
(*a*) Local structural model for α-P.O.13: ordered four-layer model with stacking sequence |*ABCD*| after optimization of the atomic coordinates by DFT-d with unit-cell parameters fixed to the values obtained by ED. This structure corresponds to a fragment with |*ABCD*| sequence embedded in a crystal with stacking disorder. View along the layers. The blue unit cell corresponds to the unit setting *C*112_1_/*g*, the black is that of the *P*1 2_1_/*c*1 setting. (*b*) Structure with stacking sequence |*ABCD*| optimized by DFT-d with free unit-cell parameters. This structure corresponds to an ordered polymorph with pure |*ABCD*| stacking. For discussion, see §3.2.11.[Sec sec3.2.11]

The analysis of the intensity distribution within the ED data revealed that the preferred local structure has a periodicity in stacking direction of *b*_L_ = 2*b*_0_ (§3.2.5). Hence, the corresponding model should consist of four layers. Only two symmetrically different four-layer sequences are possible: |*ABCD*|*ABCD*| and |*ABCB*|*ABCB*|. The ED data clearly showed a C-centred pattern; consequently, the sequence must be |*ABCD*|. This structure has a space group *C*112_1_/*g*, in which *g* stands for a glide plane with a translational vector of (*a* + *b*)/4 (Fischer & Koch, 2011 ▸)^1^. *C*112_1_/*g* is an unconventional setting of *P*2_1_/*c* [***a*** *_P_*_21/*c*_ = (−***a***_L_ + ***b***_L_)/2, ***b*** *_P_*_21/*c*_ = ***c***_L_, ***c*** *_P_*_21/*c*_ = (***a***_L_ + ***b***_L_)/2]. This unconventional setting was chosen in order to describe the model with the same unit-cell parameters as the two-layer model, except for a doubling of the *b* axis. The structure of the four-layer model is shown in Fig. 15 ▸(*a*).

The systematic extinctions for the 2_1_ axis agree with the ED data. The reflection conditions for the *g*-glide plane are *hk*0: *h* + *k* = 4*n* for |*ABCD*|, and *hk*0: *h* − *k* = 4*n* for its mirror image |*DCBA*|. These reflection conditions are not well visible in the diffraction pattern, because they are buried in the extinctions of the *C* centring of the unit cell requiring *hk*0: *h* + *k* = 2*n*.

The two-layer model as well as the four-layer model consist of molecules in the *cis* conformation. We tried to build similar models with molecules in *trans* conformation. However, the steric requirement of *trans* molecules considerably differs from that of *cis*-molecules. In all models built with the *trans* conformation, the molecules showed unreliably short intermolecular contacts. Lattice-energy minimization lead to an enlargement of the unit cell resulting in unit-cell parameters, which strongly deviated from the values obtained by ED. Hence, the molecular packing in α-P.O.13 is possible only for molecules in *cis* conformation.

#### 3.2.9. Structure refinement

We made a fast attempt to refine the different structural models against ED data using the least-squares kinematical refinement, yet soon abandoned this idea. The quality of the data was not sufficient for a quantitative treatment of the reflection intensities, possibly due to the static data collection procedure and the presence of diffuse scattering, which is known to deteriorate the data quality.

Due to the small crystal size and the presence of diffuse scattering, the refinement against X-ray powder data was not reliable, either (see below).

Therefore, the atomic coordinates were optimized by quantum-mechanical methods using density-functional theory with dispersion correction (DFT-d). These calculations were performed on the ordered two-layer model and the ordered four-layer model. The average structure could not be treated by quantum-mechanical methods, due to the disorder with overlapping atoms and an occupancy of 0.5 for all atoms. In the DFT-d calculations of the ordered models, the unit-cell parameters were fixed to the values obtained from ED. Upon optimization, the structures changed only slightly, which proves that the structural models are crystallochemically sensible.

#### 3.2.10. Comparison of simulated and experimental electron diffraction patterns

**Figure 16:**
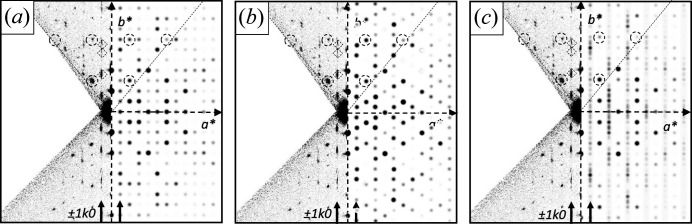
Comparison of experimental and simulated ED patterns of the [001] zone: (*a*) two-layer model, (*b*) four-layer model and (*c*) ten-layer model with the stacking sequence |*ABADABADAB*| (as shown in Fig. 14 ▸). The experimental pattern is shown on the left side, the simulated ones on the right side. The fine dashed lines denote the boundaries of the collected data wedge. The diffuse rows at ±1*k*0 are marked with bold arrows at the bottom of each pattern. The dashed circles denote three strong Bragg reflections of the average structure. The small diamonds denote intensity maxima in the diffuse row at 1*k*0.

For the structural models with two, four and ten layers, electron diffraction pattern of the [001] zone, which comprises the diffuse scattering rows, were simulated (Fig. 16 ▸). The simulated patterns were compared to the [001] section, extracted from the experimental reciprocal space volume [Fig. 8 ▸(*d*)]. Fig. 16 ▸ shows the comparison of simulated and experimental patterns. The strong Bragg reflections of the average structure are correctly reproduced by all three models (some of the Bragg reflections are marked by dashed circles in Fig. 16 ▸.) Note that the [001] zone of the four-layer model |*ABCD*| has only twofold rotation symmetry, whereas the two-and ten-layer models have 2*mm* symmetry, like the experimental pattern. For a better comparison, the *mm* symmetry was added to the four-layer model, corresponding to a mixture of |*ABCD*| and |*DCBA*| sequences, which is a quite reasonable assumption for a real crystal.

With increasing numbers of layers in the model, the diffuse rows are developing. The experimental distribution of intensities along the diffuse lines follows a certain rule: for the rods at ± 1*k*0 rod, diffuse intensity is mainly concentrated around the positions with *k* = 1.5, 3.5, 5.5, and 6.5 [see small diamonds in Figs. 16 ▸(*a*)–16 ▸(*c*)]. This distribution cannot be achieved with a two-layer model [Fig. 16 ▸(*a*)], which wrongly simulates the intensities at integer *k* values. Correspondingly, a regular *AB* stacking is not a proper representation of the structure. In contrast, the experimental intensity maxima in the ± 1*k*0 rod match the simulated intensities of the four-layer model. In addition, the intensities on the diffuse streaks at ± 3*k*0 and ± 5*k*0 are reproduced quite well. [Fig. 6 ▸(*b*)]. Hence, the four-layer model describes the diffuse scattering much better. Apparently, the |*ABCD*| (or |*DCBA*|) stacking motif is the most prominent one in the structure. The ten-layer model demonstrates that a larger periodicity of the stacking sequence leads to the formation of extended diffuse streaks, like in the experimental pattern.

#### 3.2.12. Structure validation by X-ray powder diffraction

The different structural models were validated by lattice-energy minimizations with DFT-d, and by comparison with the experimental powder pattern.

Two sets of DFT-d optimizations were performed, one with unit-cell parameters fixed to the values obtained by ED, the other one with free unit-cell parameters.

In the DFT-d calculations with unit-cell parameters from ED, the four-layer model is by 0.74 kJ mol^−1^ more favourable than the two-layer model. In the DFT-d optimizations with free unit-cell parameters, the energy difference increases to 1.86 kJ mol^−1^. Both values point to a statistical disorder with a slight preference for a local stacking with a sequence |*ABCD*| (or |*DCBA*|, respectively) over |*AB*|*AB*|.

Upon optimization with free unit-cell parameters, the two-layer structure changed only slightly, and remained orthorhombic. In the four-layer model, the symmetry (*C*112_1_/*g*) was maintained, but the angle γ changed from 90° to 83.69°, see Table 3 ▸ and Fig. 15 ▸(*b*). This change corresponds to a lateral shift of the layers by 0.78 Å per layer. Such a lateral shift is well possible in a layer structure. In a real crystal, the lateral shift ‘to the right’ within an |*ABCD*| domain would be compensated by a corresponding shift ‘to the left’ in a |*DCBA*| domain. Both, |*ABCD*| and |*DCBA*| domains are equally frequent, so that the overall crystal structure remains orthorhombic. The small energy difference between |*AB*|*AB*|, |*ABCD*| and |*DBCA*| indicates that the domains with a strict |*ABCD*| or |*DCBA*| stacking are actually quite small.

Large domains with a strict |*ABCD*| or |*DCBA*| stacking would be visible in diffraction patterns, because the local change of the γ angle from 90° to 83.69° (for |*ABCD*|) or 96.31° (for |*DCBA*|) would be visible as a splitting of the Bragg peaks. Such a splitting was not observed, neither in the ED patterns, nor in the X-ray powder patterns. Hence, the diffraction data confirm that the ordered domains with a strict |*ABCD*| (or |*DCBA*|) sequence cannot be very large.

In principle, P.O.13 could form a structure, which consists only of the lowest-energy stacking sequence |*ABCD*|. This structure would be a different polymorph. Since the unit-cell parameters, especially the angle γ, differ from that of the α phase, its powder pattern would significantly differ from the pattern of the α phase. Hence, this polymorph would be easily recognisable from its X-ray powder diffraction pattern. However, in the hundreds of powder patterns, which we recorded during the polymorph screening and in recrystallization attempts, we never observed the formation of this phase.

#### 3.2.11. Structure validation by X-ray powder diffraction

**Figure 17:**
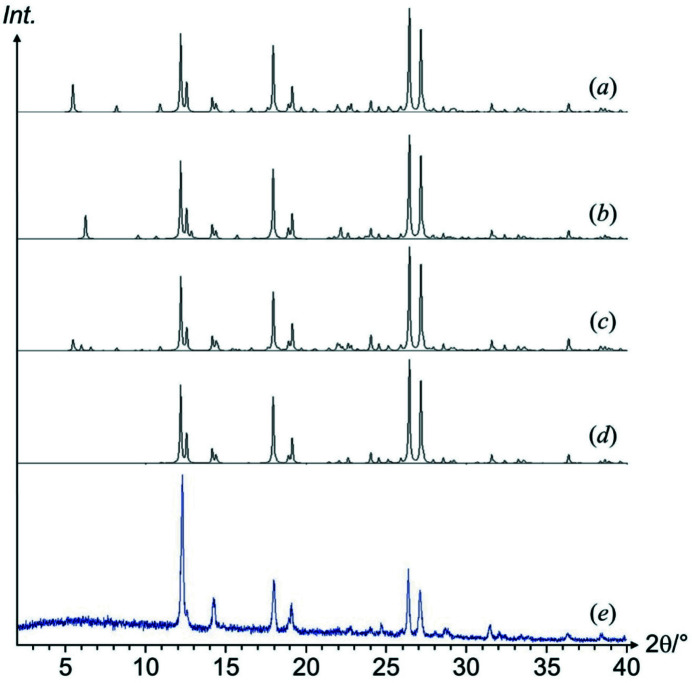
Simulated and experimental X-ray powder patterns of α-P.O.13. (*a*) Two-layer model, (*b*) four-layer model, (*c*) ten-layer model, (*d*) average structure, (*e*) experimental powder X-ray pattern.

X-ray powder patterns were calculated for the two-layer model, the four-layer model, the ten-layer model, and the average structure. All powder patterns are very similar, and match quite well the experimental powder pattern of the α phase, see Fig. 17 ▸. The main differences were found in the region below 12° 2θ. Here, the simulated powder patterns show superstructure reflections, depending on the unit cell of the structural model. The experimental powder pattern does not show any of these superstructure reflections, pointing to a statistical disorder without large ordered domains. Hence, the overall crystal structure is confirmed, but details on the preferred stacking sequence cannot be derived.

#### 3.2.13. Crystal structure of the α phase

The final structure was obtained from the combination of experimental electron diffraction data and the DFT-d calculations. Unit-cell parameters and the overall molecular packing were obtained from ED, whereas precise atomic coordinates originate from the DFT-d calculations. These structural models, the ordered two-layer and the ordered four-layer model, should be regarded as the ‘final’ structural models for the α phase of P.O.13. The two models have been deposited at the Cambridge Structural Database under the reference codes 2160710 (two-layer) and 2160709 (four-layer). The structures are shown in Figs. 12 ▸ and 15 ▸(*a*).

The crystal structure of the α phase of P.O.13 is disordered. The ordered two-layer and four-layer models give a good representation of the structure, concerning the unit-cell parameters, the molecular geometry and the arrangement of neighbouring molecules.

The two-layer model is the simplest ordered model. It is crystallochemically sensible, but does not reproduce the positions of the intensity maxima in diffuse scattering. These maxima are reproduced much better by the four-layer model. Hence, the four-layer model gives a much better representation of the actual local structure.

**Figure 18:**
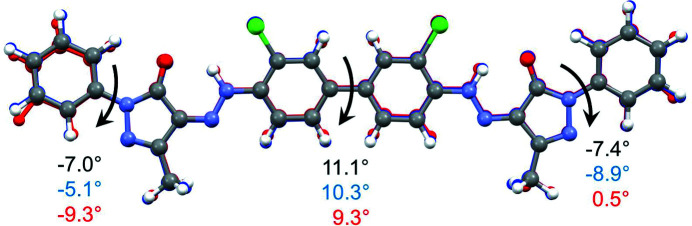
Molecular geometry in the α phase of P.O.13 (‘final’ structural models), and in the β phase (single-crystal data). The molecule of the two-layer model is drawn in element colours, of the four-layer model in blue, and of the β phase in red. The values denote the interplanar angles between the phenyl rings, and between the pyrazolone moiety and the terminal phenyl rings.

In both models, the molecular geometry is very similar to that in the β phase, see Fig. 18 ▸. The molecules adopt an overall *cis* conformation, like in the β phase.

The π–π stacking of molecules along the *c*-axis is similar to that in the β phase, but neighbouring stacks within a layer adopt a parallel packing instead of a herringbone packing, see Fig. 12 ▸.

#### 3.2.14. Why is the α phase disordered?

Simply spoken, the α phase is disordered, because the different stacking possibilities have similar lattice energies, see §3.2.11.

**Figure 19:**
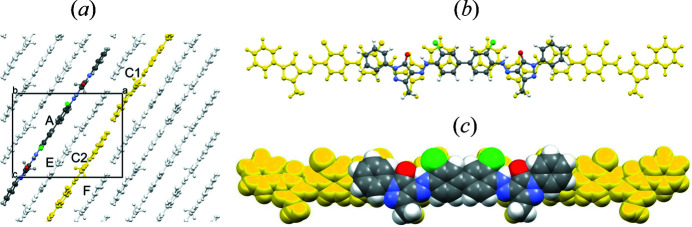
Molecular overlap in the average structure: (*a*) one ordered layer, (*b*) overlapping molecules *A*, *C*1 and *C*2, and (*c*) space-filling representation of (*b*).

For a crystallochemical explanation of the disorder, let us consider one ordered layer, see Fig. 19 ▸(*a*). The unit cell is that of the ordered two-layer model. If this layer is shifted by Δ*x* = 0.5, then the molecule *A* overlaps with the molecules *C*1 and *C*2 (in yellow). The overlapping three molecules are shown in Figs. 19 ▸(*b*) and 19 ▸(*c*). The mutual arrangement of these three molecules can also be described through a shift of the molecule by half a molecular length along the long axis of the molecule, see Figs. 19 ▸(*b*) and 19 ▸(*c*).

The molecule has the shape of a flat caterpillar. On the lower side, the methyl groups stick out. On the upper side, the chlorine atoms and the phenyl groups stick out as bumps. A shift of the molecule along its long molecular axis by half a molecular length leads to exactly the same positions of the methyl groups at the lower side, whereas at the upper side the chlorine bumps are replaced by the phenyl bumps. Hence, the combination of molecules *C*1 and *C*2 reproduces the shape of molecule *A* almost exactly. In other words: the chain of molecules along their long direction has a shape with a periodicity of half a molecular length. This ‘pseudosymmetrical’ molecular shape (Hörnig *et al.*, 1993 ▸) allows the layers to be shifted by Δ*x* = 0.5, with almost no change in the shape of the surface of the layer. The layer surface has a periodicity of *a*/2 instead of *a*. Since there are no hydrogen bonds and no strong electrostatic interactions between the layers, any subsequent layer can adopt two lateral positions which differ by *a*/2.

Figs. 19 ▸(*a*) and 19 ▸(*b*) suggest that an entire chain of molecules [molecules *C*1 + *C*2 in Fig. 19 ▸(*a*)] might be shifted along the chain direction by half a molecule, without disturbing the interactions with the neighbouring layers. However, such a chain shift would change the interactions to neighbouring chains within the layer [*e.g.*, the van der Waals and Coulomb interaction of molecule *C*2 with its neighbours *E* and *F* in Fig. 19 ▸(*a*)]. This shift corresponds to a stacking disorder of the chains within the layer. Such a disorder would cause diffuse streaks parallel to [201] and 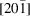 . Indeed, the experimental [010] zone pattern [Fig. 8 ▸(*f*)] contains faint diffuse streaks in these directions.

Why does the β phase not show any disorder? The reason is apparent from Fig. 19 ▸(*a*): in the β phase, molecule *C*1 has a different orientation [see Fig. 6 ▸(*c*)], hence the layer does not have a pseudosymmetric surface like in the α phase.

#### 3.2.15. On the morphology of the α phase

In the crystal structure of the α phase the weakest intermolecular interactions are in the **a** direction. There are only van der Waals interactions between the terminal phenyl rings, see Fig. 19 ▸(*a*). Correspondingly, the crystal morphology is a platelet parallel to (100), which explains the observation from TEM and ED.

### 3.3. Further crystal phases

In total, seven crystal phases are known for P.O.13 (Bekö *et al.*, 2014 ▸). The α phase is formed directly in the synthesis. The β phase, which is the thermodynamically more stable, appears after recrystallization from most solvents. The β phase is also formed if P.O.13 is dissolved in amino­ethanol and precipitated with methanol, ethyl acetate, acetone, butanone or toluene. Crystallization from 2-amino­ethanol/1,4-dioxane at room temperature leads to two different phases, δ or ɛ_1_, depending on the crystallization conditions. When a solution of P.O.13 in 2-amino­ethanol is treated with liquid dioxane, the δ phase is obtained, which is a solvate. In contrast, adding the dioxane slowly *via* gas phase diffusion leads to the ɛ_1_ phase, which turned out to be a dioxane solvate hydrate. The ɛ_2_ phase is formed upon recrystallization in morpholene. It appears to be a morpholene solvate. It could be obtained only as mixtures with the δ and/or β phases. The ζ phase is obtained by recrystallization in 1-chloro­naphthalene. The investigated powder of the ζ phase probably contains traces of other phases. An amorphous phase of P.O.13, called η-phase is formed by treating P.O.13 with concentrated sulfuric acid, followed by precipitation with water vapours.

The phases α, β, δ and η are solvent-free, according to TGA, DSC, IR and elemental analyses. Phases ɛ_1_ and ɛ_2_ are solvates. The composition of the ζ phase could not be determined (Probably the sample was not pure).

**Figure 20:**
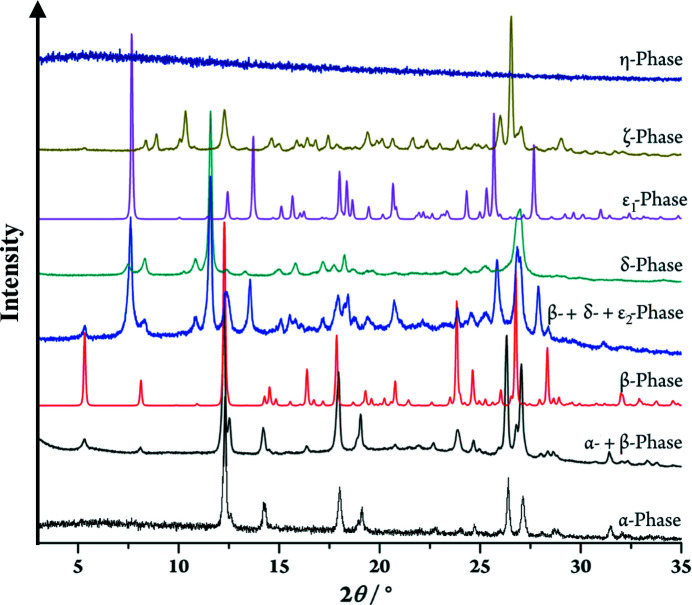
X-ray powder diffractograms of P.O.13 polymorphs.

The powder X-ray diffraction patterns of all phases are shown in Fig. 20 ▸.

The low crystallinity of the phases δ and ζ prevented the determination of their crystal structures. The η phase is amorphous.

### 6.3. Crystal structure of the phases ɛ_1_ and ɛ_2_

The phase ɛ_1_ is a solvate of P.O.13 with dioxane and water in a ratio of P.O.13: dioxane: H_2_O = 1:2:1. The crystal structure of this phase was determined by single-crystal X-ray analysis (see Table 2 ▸). The crystal was a thin needle. The final *R* values were poor, mainly due to heavily disordered solvent molecules. The compound crystallizes in the monoclinic space group *C*2/*c*, *Z* = 4, *Z*′ = ½. The P.O.13 molecule and the water molecule are situated on crystallographic twofold axes, the dioxane molecule in the general position. Surprisingly, this phase of P.O.13 also adopts a *cis* conformation, like in the α and β phases, in contrast to all other bi­phenyl-hydrazone pigments. The dihedral angle φ_1_ of the central bi­phenyl moiety is 22.4°. Despite this deviation from planarity, the π–π stacking of neighbouring molecules is very similar to those in α and β phases.

The terminal phenyl rings are almost coplanar with the pyrazole rings (φ_2_ = φ_3_ = 1.22°).

**Figure 21:**
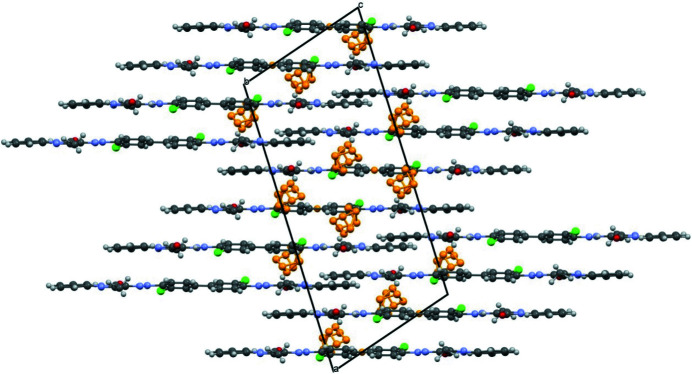
Molecular packing in the ɛ_1_ phase of P.O.13 (dioxane disolvate mono-hydrate). The water molecules and the disordered dioxane molecules are drawn in orange. View direction 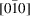 .

The molecules arrange in layers. The voids in the layers are filled by dioxane and water molecules. The dioxane molecules are orientationally disordered. The water molecule donates hydrogen bonds to two dioxane molecules. The P.O.13 molecule forms only intramolecular hydrogen bonds, but no intermolecular hydrogen bond to the dioxane or water molecules. The packing is shown in Fig. 21 ▸.

The ɛ_2_ phase is a morpholene disolvate. It could not be obtained as a pure phase, but only as a mixture with the phases β and/or δ. Single crystals could not be grown. However, the high similarity of the powder patterns of the ɛ_1_ and ɛ_2_ phases suggests that the crystal structure of the ɛ_2_ phase is very similar to that of the ɛ_1_ phase. Apparently, the two dioxane molecules are just replaced by two morpholene molecules, which is easily possible, because the morpholene molecule deviates from a dioxane molecule only by an exchange of O versus NH; hence their steric requirements are similar. Whether the morpholene disolvate again contains a water molecule, remains unknown.

## 4. Conclusion

The industrially relevant α phase of P.O.13, which is used to print covers of *Acta Crystallographica Section C*, is a nanocrystalline powder with severe stacking disorder. All attempts to improve the crystallinity and to solve the structure by powder or single-crystal X-ray diffraction had failed. We, therefore, turned to electron diffraction. The electron diffraction data contained intense diffuse scattering, which prevented a classical structure solution. Yet, a careful analysis of the positions of the Bragg reflection and of the diffuse scattering, and a certain similarity of the unit-cell parameters of the α phase with the previously determined β phase allowed us to solve the crystal structure with paper and pencil. Different structural models were built, consisting of two, four and ten layers. These models were subsequently validated by lattice-energy minimization with DFT-d. The structural model with four layers gave a quite good fit to the experimental electron diffraction data, including the main features of the diffuse scattering.

P.O.13 is the first di­aryl pigment which exhibits a *cis* conformation of the central bi­phenyl fragment. This conformation is found in all four phases (α, β, ɛ_1_ and ɛ_2_), although the individual molecule can adopt *cis* or *trans* conformations with similar energies, and all other di­aryl pigments adopt the *trans* conformation in the solid state.

We finally would like to emphasize the power of non-standard approaches for crystal structure analysis, which are rarely used nowadays with the availability of automated and standardized procedures. Yet manual approaches sometimes can be the only choice for complex crystallographic problems.

The most difficult problems are the most interesting ones.

## Supplementary Material

Crystal structure: contains datablock(s) I. DOI: 10.1107/S2052520623000720/je5050sup1.cif

Structure factors: contains datablock(s) I. DOI: 10.1107/S2052520623000720/je5050Isup2.hkl

Supporting information file. DOI: 10.1107/S2052520623000720/je5050sup3.pdf

Supporting information file. DOI: 10.1107/S2052520623000720/je5050Isup4.cml

CCDC reference: 2160709

## Figures and Tables

**Table 1:** Unit-cell parameters of α-P.O.13 determined using electron diffraction of four crystals (original unit-cell setting)

Crystal No.	1	2	3	4	Final values
Total tilt range (°)	50	86	86	66	–
*a*_o_ (Å)	16.2	16.9	16.2	16.2	16.1 (3)
*b*_o_ (Å)	14.4	14.4	14.5	14.3	14.5 (2)
*c*_o_ (Å)	12.2	12.3	12.4	12.2	12.3 (1)
α_o_ (°)	89.7	89.4	90.6	89.8	90
β_o_ (°)	89.7	90.5	91.1	88.0	90
γ_o_ (°)	89.8	91.4	88.9	88.7	90

**Table 2:** Crystallographic data of the β and ɛ_1_ phases of P.O.13 determined by single-crystal X-ray analyses

	β phase	ɛ_1_ phase
Crystal data		
Chemical composition	P.O.13	P.O.13·2 dioxane·H_2_O
Chemical formula	C_32_H_24_Cl_2_N_8_O_2_	C_32_H_24_Cl_2_N_8_O_2_·2C_2_H_4_O_2_·H_2_O
CSD entry No.	2160372	2160371
*M*_r_	623.49	831.65
Crystal system	Orthorhombic	Monoclinic
Temperature (K)	296 (2)	296 (2)
Space group	*Pbca* (No. 61)	*C*2/*c* (No. 15)
*Z*, *Z*′	8, 1	4, ½
*a* (Å)	33.137 (9)	26.6953 (13)
*b* (Å)	14.425 (4)	12.9057 (6)
*c* (Å)	12.127 (3)	12.2307 (5)
β (°)	90	106.665 (2)
*V* (Å^3^)	5797 (3)	4036.8 (3)
Crystal habit	Needle	Needle
Crystal size (mm)	0.3 × 0.05 × 0.05	0.1 × 0.01 × 0.01

Data collection		
Diffractometer	Bruker AXS three-circle goniometer	Bruker AXS three-circle goniometer
Wavelength (Å)	1.54178	1.54178
θ_max_ (°)	49.56	69.64

Refinement		
No. of measured reflections	18093	10810
No. of unique reflections	2897	3546
*R*_int_	0.0918	0.0368
No. of parameters	477	324
No. of restraints	0	0
*wR*(*F*)	0.0776	0.2329
*R*[*F* > 2σ(*F*)]	0.0576	0.0700
*S*	1.074	0.974
Δρ_max_, Δρ_min_ (e^−^Å^3^)	0.143, −0.147	0.498, −0.248

**Table 3:** Unit-cell parameters of α and β P.O.13 determined by different methods Abbreviations: SC-XRD = single-crystal X-ray diffraction, ED = electron diffraction, DFT-d = density functional theory with dispersion correction. The original unit-cell parameters *a*_0_, *b*_0_, *c*_0_ from ED are those given in the column ED/DFT-d for the ordered two-layer model.

Phase	β	α					
Model		Average structure	Ordered two-layer model			Ordered four-layer model	
Method	SC-XRD	ED	ED/DFT-d†	DFT-d‡	XRPD	ED/DFT-d†	DFT-d‡
CSD entry No.	2160372	–	2160710	–		2160709	–
Space group	*Pbca*	*Pbcn*	*Pbc*2_1_	*Pbc*2_1_	*Pbc*2_1_	*C*112_1_/*g* §	*C*112_1_/*g* ¶
*Z*	8	2	4	4	4	8	8
*a* (Å)	33.137 (9)	8.1 (3)	16.1 (3)	15.9644	16.142	16.1 (3)	16.1273
*b* (Å)	14.425 (4)	14.5 (2)	14.5 (2)	14.2957	14.405	29.1 (4)	28.3484
*c* (Å)	12.127 (3)	12.3 (1)	12.3 (1)	12.5389	12.368	12.3 (1)	12.5362
α (°)	90	90	90	90	90	90	90
β (°)	90	90	90	90	90	90	90
γ (°)	90	90	90	90	90	90	83.6858
*V* (Å^3^)	5797 (3)	1436 (30)	2871 (60)	2861.65	2876	5742 (120)	5696.54

† Final structural models for the α phase. Unit-cell parameters from ED, atomic coordinates optimized by DFT-d.

‡ Unit-cell parameters and atomic coordinates from DFT-d.

§ Standard setting: *P*2_1_/*c*, *Z* = 4, *a* = *c* = 16.6 (3) Å, *b* = 12.3 (1) Å, β = 58.1 (9)°, α = γ = 90°, *V* = 2871 Å^3^.

¶ Standard setting: *P*2_1_/*c*, *Z* = 4, *a* = 15.5174 Å, *b* = 12.5362, *c* = 17.0608 Å, β = 59.1173°, α = γ = 90°, *V* = 2848.27 Å^3^.

## Footnotes

1 This glide plane is denoted as *g*, not as *d*, because a *d* glide plane with a translational vector of (*a* + *b*)/4 requires the presence of a second ’*d*’ glide plane with a translation vector of (*a* − *b*)/4, which is absent in this model.
