# Supplementary material for: Analysis of diffuse scattering in electron diffraction data for the crystal structure determination of Pigment Orange 13, C32H24Cl2N8O2
Source: Acta Crystallogr B Struct Sci Cryst Eng Mater. 2023 Feb 24;79(Pt 2):122–37. doi: 10.1107/S2052520623000720 (PMC10088482; doi:10.1107/S2052520623000720)
Supplement: Supplementary file 3 [file b-79-00122-sup3.pdf]

STRUCTURAL SCIENCE
CRYSTAL ENGINEERING
MATERIALS

Volume 79 (2023)

Supporting information for article:

Analysis of diffuse scattering in electron diffraction data for the crystal structure determination of Pigment Orange 13, C32H24Cl2N8O2

Tatiana E. Gorelik, Sàndor L. Bekö, Jaroslav Teteruk, Winfried Heyse and Martin U. Schmidt

1.

2. CSD search on the interplanar angle in pyrazolone pigments

The interplanar angle between the pyrazolone moiety and the terminal phenyl ring in pyrazolone hydrazone pigments was investigated by a search in the Cambridge Structural Database.

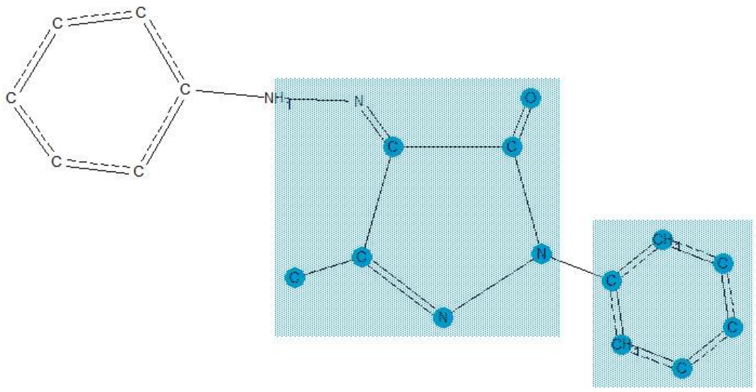

Figure S1 CSD search fragment. The interplanar angle between the two planes shown in blue is denoted as phi2 .

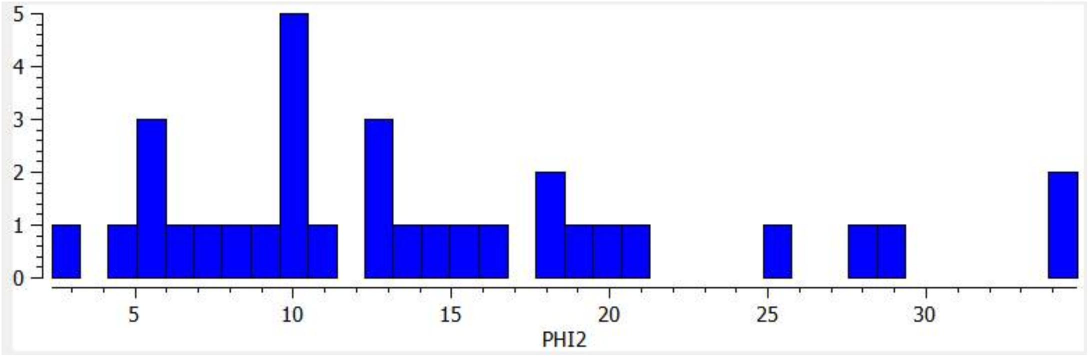

Figure S2 Histogram of the interplanar angle phi2.

Remark: The "outlier" with a phi2 angle of 34-35° (1 compound with two CSD entries) is not an unusual molecule, but a typical pyrazolone pigment (Figure S3). From this observation, we conclude that the interplanar angle between the pyrazolone fragment and the phenyl group can adopt all values between 0° and 35°.

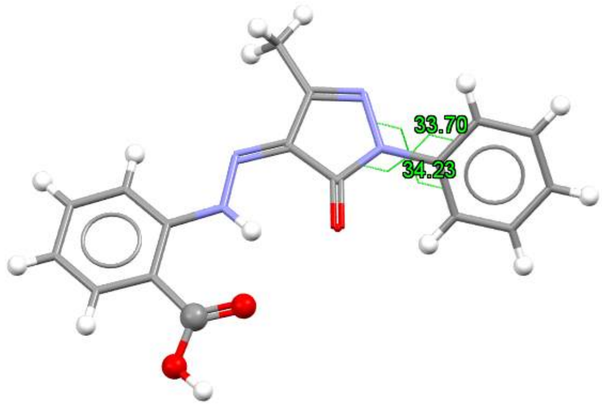

Figure S3 Pyrazolone compound with a phi2 angle of about 34°. (The values are the NNCC and CNCC torsion angles).
